# The Use of Teledermatology for Skin Cancer Referrals: A Retrospective Study in a Single Tertiary Centre

**DOI:** 10.7759/cureus.68284

**Published:** 2024-08-31

**Authors:** Arvindh Sekaran, Evangelia Vetsiou, Ashka Shah, Andre Khoo

**Affiliations:** 1 Internal Medicine, Addenbrooke's Hospital, Cambridge University Hospitals NHS Foundation Trust, Cambridge, GBR; 2 Dermatology, Addenbrooke's Hospital, Cambridge University Hospitals NHS Foundation Trust, Cambridge, GBR; 3 General Medicine, Addenbrooke's Hospital, Cambridge University Hospitals NHS Foundation Trust, Cambridge, GBR

**Keywords:** diagnostic concordance, skin cancer, dermatology, telemedicine, teledermatology

## Abstract

Introduction: Teledermatology utilises telecommunications technology to deliver dermatological care remotely, incorporating live video consultations, store-and-forward systems, and hybrid approaches. It is particularly valuable in underserved or remote areas with limited access to dermatologists. Reported benefits include reduced face-to-face consultations for benign lesions, leading to increased capacity for severe cases, improved access for rural patients, and enhanced satisfaction among clinicians and patients. The COVID-19 pandemic accelerated the adoption of teledermatology, integrating it into the National Health Service (NHS) framework for managing referrals and ensuring continuity of care. This study examines the outcomes of two-week wait referrals for suspected skin cancer, focusing on diagnostic concordance between teledermatology and histopathology.

Materials and methods: The study was conducted at Addenbrooke's Hospital, part of Cambridge University Hospitals, via a retrospective review of patient records from November 2022 to May 2023. Inclusion criteria were all patients referred by their general practitioner (GP) under the two-week wait for suspected skin cancer pathway. Data collected included patient demographics, waiting times, clinical and histological diagnoses, and patient re-referrals for the same problem. The primary objective was to assess diagnostic concordance between the clinical diagnosis from teledermatology and histopathology. Secondary objectives included accuracy of lesion site description, patient waiting times, and computed time savings from the use of teledermatology.

Results: The study covered 71 patients (34 males, 37 females) aged 19-87 years (mean: 59.63), with Fitzpatrick skin I-III predominating. A total of 110 individual lesions were assessed, and 46 required surgical management. Clinical and histological concordance was 62%, with 100% accuracy for basal cell carcinoma (BCC) and melanoma. The service saved 10 hours of consultant time and reduced the need for 62 initial face-to-face consultations. Lesion site documentation had a 73% correlation between GPs and dermatologists. Diagnoses varied widely between GPs and dermatologists, with a 31% concordance.

Conclusion: Our study shows that teledermatology is a safe and effective method for managing two-week wait referrals for suspected skin cancer, reducing footfall, and saving time and costs for both clinicians and patients. While there are limitations, the usage of teledermatology allows increasingly limited capacity for face-to-face consultations to be reserved for high-risk patients. Further studies in different regions should explore teledermatology's utility across diverse demographics, particularly to address healthcare disparities for those with darker skin tones.

## Introduction

Teledermatology, a branch of telemedicine, leverages telecommunications technology to provide dermatological care from a distance. This modality encompasses various services, including live video consultations (synchronous), store-and-forward systems (asynchronous), and hybrid approaches.

The benefits of a teledermatology service include reducing the number of face-to-face consultations when faced with restrictions in physical capacity, thereby reserving this capacity for the assessment of more high-risk patients face-to-face. Additionally, it reduces the need for travel, thereby saving time and costs for patients and the healthcare system [1]. Teledermatology has been shown to increase satisfaction among clinicians and patients [2,3]. It has the potential to enable prompt assessment of patients referred with both benign and malignant skin lesions. Studies have demonstrated that teledermatology can achieve diagnostic accuracy comparable to in-person consultations, making it a reliable alternative [4-10].

The COVID-19 pandemic has significantly accelerated the adoption of teledermatology worldwide. The necessity for social distancing and the constraints on face-to-face medical consultations have led to a rapid shift towards remote healthcare solutions. During the pandemic, teledermatology emerged as a crucial tool to maintain continuity of care while minimising the risk of viral transmission. For example, a study highlighted a dramatic increase in teledermatology consultations in the early months of the pandemic, showcasing its vital role in healthcare delivery during such unprecedented times [11].

In the United Kingdom, teledermatology has been integrated into the National Health Service (NHS) with the support of the British Association of Dermatologists (BAD), together creating a roadmap on how to deliver clinical pathways for teledermatology, especially following the pandemic [12]. The NHS has adopted teledermatology to manage patient referrals, allowing general practitioners (GPs) to send images of skin conditions to dermatologists for evaluation via the NHS e-Referral Service (e-RS) and commercial teledermatology platforms. Within secondary care, dermatology departments have adopted teledermatology by arranging for professional (either a nurse or medical photographer-led) capture of images for clinician review. This store-and-forward method has been particularly effective in triaging cases, thereby prioritising urgent conditions for in-person consultations and managing less critical cases remotely [13,14]. There is less impetus for the uptake of video consultations, particularly with the easing of pandemic restrictions. The medium is restrictive in terms of the quality of images delivered, and there is little benefit compared to either store-and-forward or a conventional face-to-face consultation. Teledermatology services in the UK are supported by various digital platforms that ensure the secure transmission of medical data and images. These platforms are integrated with electronic health records (EHRs), facilitating seamless communication and continuity of care.

Our centre has used teledermatology since 2019 for the management of patients referred with skin lesions, and the service undergoes regular audits to ensure compliance with the BAD Teledermatology Service Standards. In 2022, the service expanded to include patients referred for suspected skin cancer on the NHS Suspected Cancer ("two-week wait") pathway. Our study looks at these two-week wait referrals for suspected skin cancer to our teledermatology service. It describes the outcomes of these referrals, especially regarding the diagnostic concordance between teledermatology diagnoses and histopathological diagnoses in biopsied lesions.

## Materials and methods

Our study involved a single dermatology department providing a teledermatology service in Addenbrooke's Hospital, part of the Cambridge University Hospitals NHS Foundation Trust. The hospital is located in the city of Cambridge and serves a mixed urban and rural population. We conducted a retrospective review of patient records over a seven-month period from November 2022 to May 2023. The inclusion criteria encompassed all patients referred by their GP to the teledermatology service as part of a two-week wait referral for suspected skin cancer.

**Figure 1:**
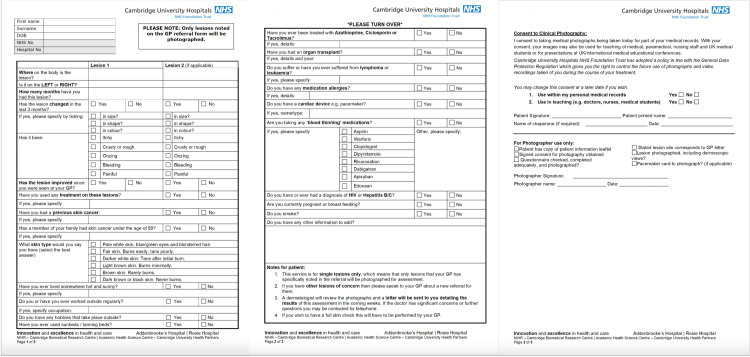
Community clinic proforma

Patients referred under this scheme are seen in a community diagnostic hub, where they complete a proforma detailing their medical history and chief complaint (Figure 1). Following this, a medical photographer takes macroscopic and dermoscopic photographs of the target lesion(s). Subsequently, a dermatologist analyses these images, and an outcome letter is produced for the patient and referring clinician.

The variables collected and analysed included the age and gender of the patient, date of referral, date of medical photography, date of teledermatology review, diagnosis made by the GP and dermatologist, onward referral for lesions if applicable, histology reports for lesions that were biopsied, and evidence of GP re-referral for the same lesion within an eight-month period following discharge.

The primary objective was to assess diagnostic concordance between the clinical diagnosis made by the dermatologist during the teledermatology review and the final histopathological diagnosis. This concordance is calculated as the total number of biopsied lesions with histology that supports the clinical diagnosis divided by the total number of biopsied lesions. Secondary objectives included the lesion site concordance between GP and dermatologist, the waiting times between initial referral and teledermatology review, and the amount of time saved by using the teledermatology service.

## Results

Primary objectives

Our study included a total of 71 patients, comprising 34 males and 37 females, with ages ranging from 19 to 87 years (mean age: 59.63). Seventy of these patients had skin types I-III on the Fitzpatrick scale. A total of 87 lesions were referred by GPs, with two lesions not assessed via the teledermatology service due to prior consultations with plastic surgeons. Consequently, 110 lesions were assessed, including 25 additional lesions identified at the time of medical photography, either at the patient's request, the photographer's concerns regarding index lesion localisation, or ambiguity on the referral.

**Figure 2:**
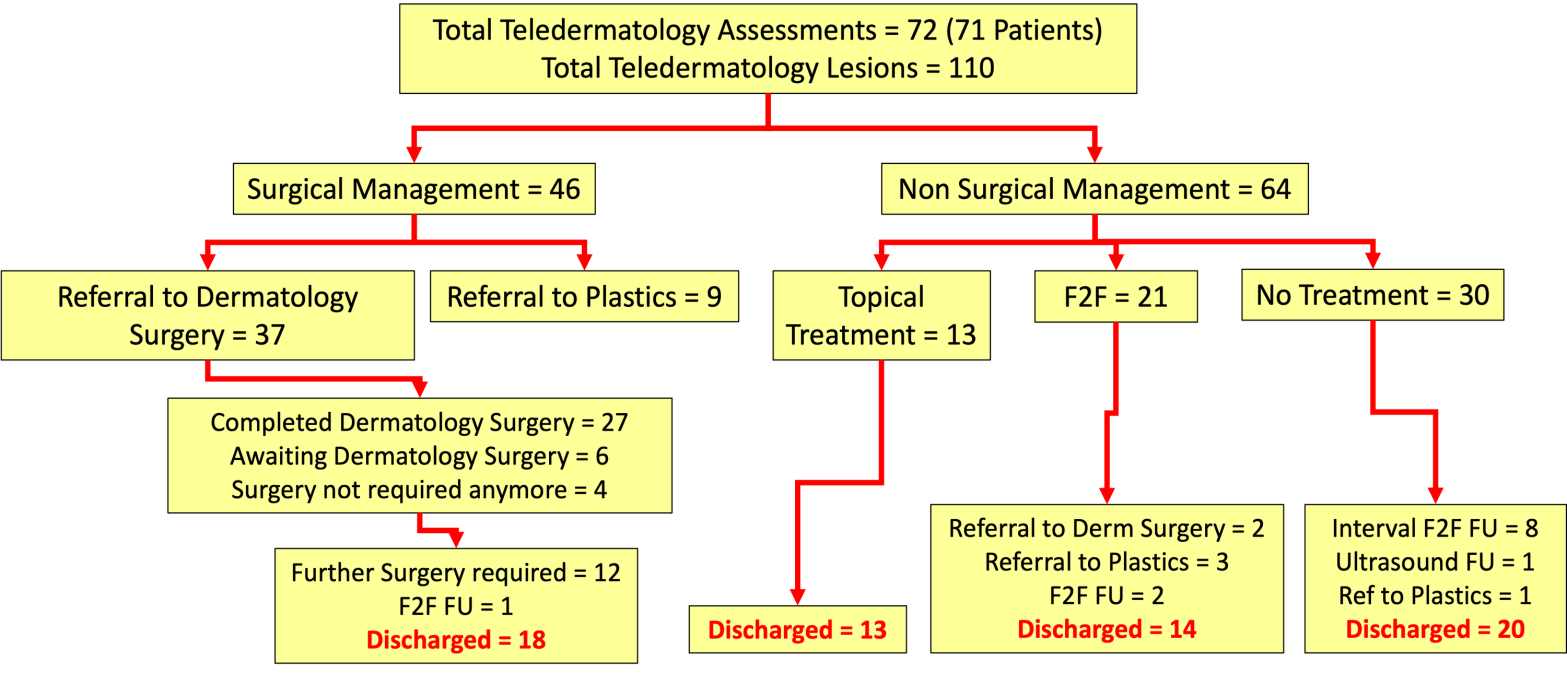
Patient pathways after assessment in the teledermatology service F2F: face-to-face; FU: follow-up

Of the 110 lesions assessed, 46 required surgical management, and 64 were managed non-surgically. Of those requiring surgery, 37 were treated by the dermatology team, and nine were referred to plastic surgery. Of the remainder, 30 were discharged without requiring treatment, and a further 13 were to have topical treatment via primary care. A total of 21 lesions had follow-up face-to-face consultations due to ambiguity with the photograph and/or for full skin checks. Out of the 30 who did not require treatment, eight had interval face-to-face follow-ups, one had an ultrasound follow-up, and 20 were discharged. Overall, 65 lesions were discharged, with the other 45 requiring further follow-up/surgery or being taken over by the plastic surgery service (Figure 2).

The overall clinical and histological concordance was 62%, with basal cell carcinoma (BCC) confirmed histologically in 100% of cases (7/7), squamous cell carcinoma (SCC) in 67% (2/3), and melanoma in 100% (1/1) (Table 1). Five lesions, which were clinically felt more likely to be benign prior to biopsy, were found to be malignant: two seborrhoeic keratoses were found to be a BCC and SCC, two Bowen's disease lesions were found to be SCCs, and one pyogenic granuloma was found to be a melanoma. The discordant SCC was found to be a benign keratosis. These results should be interpreted in the context of local NHS commissioning policy: no surgery was to be carried out for benign lesions (even if symptomatic), and all lesions that underwent biopsy had at least some degree of diagnostic uncertainty and possibility of malignancy.

**Table 1:** Diagnostic concordance between the clinical diagnosis and histology

Dermatology Clinical Diagnosis	Number of Lesions	Number Confirmed by Histology	Concordance
Benign lesions	18	13	72%
Basal cell carcinoma	7	7	100%
Other—malignant	6	0	0%
Concerning melanocytic lesion	6	2	33%
Squamous cell carcinoma	3	2	67%
No specific diagnosis	1	1	100%
Melanoma	1	1	100%

The teledermatology service resulted in significant savings in both clinician time and physical capacity within the department, reducing the need for 62 initial face-to-face consultations, translating into approximately five x four-hour clinic sessions or physical capacity created and 10 hours of consultant time as teledermatology assessments were allocated less time per patient compared to a face-to-face clinic review.

Secondary objectives

There was a good correlation between the lesion sites identified by GPs and dermatologists in 73% (62/85) of cases. However, 21% (18/85) showed poor correlation, 3.5% (3/85) had incorrect sites listed, and 2.5% (2/85) had no site indicated on the referral. Examples of site documentation can be seen in Table 2. Lesion size was measured by GPs in 86% (73/85) of cases and by dermatologists in 92% (101/110) of cases.

**Table 2:** Examples of differences in site documentation

General Practitioner Site	Dermatologist Site
Left upper back	Right upper back
Left lower leg	Right posterior lower leg
Right upper back	Left upper back
Scalp	Right vertex scalp
Back	Left lateral mid back

Dermatologists diagnosed a range of conditions, including benign lesions, seborrhoeic keratosis, benign melanocytic naevi, BCC, other malignant conditions such as Merkel cell carcinoma, concerning melanocytic lesions such as dysplastic naevi, SCC, no specific diagnosis, and melanoma (Table 3). In contrast, GP diagnoses were largely skewed towards SCC, with 27 cases having no diagnosis indicated, followed by melanoma, BCC, other benign conditions, and concerning melanocytic lesions (Table 4). Overall, there was a 31% (18/58) concordance between GP and dermatologist diagnoses, with 32% (27/85) of cases having no diagnosis indicated by the GP.

**Table 3:** Summary of diagnoses by dermatologists

Dermatology Clinical Diagnosis	Number of Lesions
Other - benign	33
Seborrhoeic keratosis	21
Benign melanocytic naevus	21
Basal cell carcinoma	15
Other - malignant	8
Concerning melanocytic lesion	7
Squamous cell carcinoma	3
No specific diagnosis	1
Melanoma	1

**Table 4:** Summary of diagnoses by general practitioners

General Practitioner Clinical Diagnosis	Number of Lesions
Squamous cell carcinoma	30
None indicated	27
Melanoma	18
Basal cell carcinoma	6
Other—benign	3
Concerning melanocytic lesion	1

For lesions referred as SCC, the final clinical diagnosis included BCC, actinic keratosis, Bowen's disease, SCC versus other differentials, benign keratosis, and viral warts (Table 5). Melanoma referrals resulted in the diagnoses of seborrhoeic keratosis, atypical melanocytic lesions, other benign melanocytic lesions, haemangiomas, junctional naevi, solar lentigo, blue naevus, non-specific diagnoses, melanoma, and lentigo maligna (Table 6).

**Table 5:** The final clinical diagnoses for lesions referred as squamous cell carcinoma

General Practitioner Squamous Cell Carcinoma -> Final Clinical Diagnosis	Number of Diagnoses
Basal cell carcinoma	7
Actinic keratosis	6
Bowen's disease	4
Versus squamous cell carcinoma, e.g. Bowenoid actinic keratosis/pyogenic granuloma/ulcer	4
Squamous cell carcinoma	3
Seborrhoeic/benign keratosis	3
Viral warts	3
Total	30

**Table 6:** The final clinical diagnoses for lesions referred as melanoma

General Practitioner Melanoma -> Final Clinical Diagnosis	Number of Diagnoses
Seborrhoeic/benign keratosis	3
Atypical melanocytic lesion	3
Other benign melanocytic lesion	3
Haemangioma	2
Junctional naevus	2
Solar lentigo	1
Blue naevus	1
No specific diagnosis	1
Melanoma	1
Lentigo maligna	1
Total	18

One patient was re-referred by a GP involving an enlarging BCC on the cheek who was already awaiting Mohs surgery, having previously seen the teledermatology service.

Only 10 out of 71 patients required a face-to-face appointment after the teledermatology review, leading to various treatments and subsequent discharge (Table 7). Of these face-to-face follow-ups, four additional incidental lesions were identified by the dermatologist for two patients, which were all benign.

**Table 7:** Outcomes for face-to-face appointments

Number of Patients | Face-to-Face Assessment	Outcome
2 - Plastics referral	Discharge
2 - Cryotherapy	Discharge
2 - Topical treatment	Discharge
1 - Excision	Discharge
1 - Full skin check	Discharge
1 - Lesion check	3-month follow-up + excision + discharge

The average time taken from the date of referral to the review in the teledermatology clinic was 19 days.

## Discussion

As part of teledermatology quality standards set out by NHS England, ensuring our teledermatology service is serving our local population's needs is paramount for its success. In patients with lesions identified by the teledermatology clinic and subsequently biopsied, we observed a diagnostic concordance between the clinical and histological diagnoses to be 62%. This value is consistent with previous reports of diagnostic concordance when using the store-and-forward technique for dermatology [10]. The true concordance value may be higher when including the benign lesions that were discharged directly from the teledermatology clinic without the need for biopsy and were not re-referred. Although five benign lesions were found to be malignant, they were accurately referred for biopsy, and the teledermatology service was successful in capturing all aggressive malignant lesions, especially melanoma, in a timely manner, thus providing a high level of patient safety. Additionally, this service saved the equivalent of 10 hours of consultant dermatologist time, which can be devoted to conditions that can only be managed in-person. It also reduced travel/parking costs and journey times for 62 patients, which is a significant positive variable to consider.

One of the main limitations of the service was when additional lesions that were not identified or referred by the GP were photographed, which contributed to patients being called for a face-to-face appointment and added to the footfall for the dermatology service. This becomes especially difficult for the medical photographer to decline when requested by the patient if they have travelled a significant distance for the photography appointment, are frail, and/or find it hard to arrange a GP appointment, which is a growing problem across the UK post-pandemic [15]. Another limitation to highlight is that our service's utility is not representative of those of richer skin tones (types four to six on the Fitzpatrick scale), given only one out of 71 patients fits this category; hence, the results of our study can only be reliably considered by others when using teledermatology for patients with lighter skin types. However, skin cancer is also disproportionately present in this cohort. This could present a hurdle in larger cities where there is a larger proportion of ethnic minorities with darker skin types and for whom a face-to-face appointment may be safer than teledermatology to provide an accurate diagnosis [16,17]. The final limitation is the way concordance is established. This may differ between studies as benign skin lesions encompass various lesions, which may not be strictly concordant with the histological diagnosis. However, this makes little difference clinically as the lesions are still classed as benign/harmless.

## Conclusions

Our study demonstrates that our teledermatology service is safe and effective in managing two-week wait referrals for suspected skin cancer. It significantly reduces footfall for both clinicians and patients, saving time and associated financial costs. While recognising the service's limitations, it allows for reserving face-to-face consultations for those who most require a full skin examination. Further studies across hospitals in different regions should assess the utility of teledermatology services in a wider range of demographics, especially to reduce healthcare inequality for those with darker skin tones, for whom teledermatology may not always be effective.
